# Complete genome sequence of an extensively drug resistant (XDR) *M. morganii* SMM01 isolated from a patient with urinary and fecal incontinence

**DOI:** 10.1186/s12863-021-00982-3

**Published:** 2021-08-16

**Authors:** Pachi Pulusu Chanakya, Balaram Khamari, Manmath Lama, Arun Sai Kumar Peketi, Prakash Kumar, Valakunja Nagaraja, Eswarappa Pradeep Bulagonda

**Affiliations:** 1 Department of Biosciences, AMR Laboratory, Sri Sathya Sai Institute of Higher Learning, Puttaparthi, India; 2 Department of Microbiology, Sri Sathya Sai Institute of Higher Medical Sciences, Prasanthigram, Puttaparthi, India; 3 Department of Microbiology and Cell Biology, Indian Institute of Science, Bengaluru, India; 4 Jawaharlal Nehru Centre for Advanced Scientific Research, Jakkur, Bengaluru, India

**Keywords:** *M. morganii*, Extensively drug resistant (XDR), Hybrid sequencing, Complete genome and antimicrobial resistance (AMR)

## Abstract

**Objective:**

*M. morganii* is a gram-negative, non-lactose fermenting and an opportunistic pathogen frequently associated with nosocomial infections. Although first isolated in 1906 from a pediatric fecal sample, not many *M. morganii* isolates have been sequenced. The objective of this work is to determine the complete genome sequence of an XDR *M. morganii* strain (SMM01) isolated from the urine of a patient with urinary and fecal incontinence and to characterize its antimicrobial resistance profile.

**Data description:**

Here, we report the complete genome sequence of *M. morganii* SMM01 generated from the hybrid assembly of Illumina HiSeq X and Nanopore MinION reads. The assembly is 100% complete with genome size of 39,30,130 bp and GC content of 51%. Genomic features include 3617 CDS, 18 rRNAs, 78 tRNAs, 4 ncRNAs and 60 pseudogenes. Antimicrobial resistance profile was characterized by the presence of genes conferring resistance to aminoglycosides, β-lactams, fluoroquinolones, chloramphenicol, and tetracyclines. Secondary metabolite biosynthetic gene clusters like NRPS, T1PKS, thiopeptide, beta-lactone, and bacteriocin were identified. The genome data described here would be the first complete genome of an Indian *M. morganii* isolate providing crucial information on antimicrobial resistance patterns, paving the way for further comparative genome analyses.

## Objective

*M. morganii* is a gram-negative, facultative, non-lactose fermenting bacterium belonging to the tribe *Proteae* of *Enterobacteriaceae* family. This opportunistic pathogen was first reported in 1906 from a pediatric fecal sample [1]. *M. morganii* is often encountered among postoperative, immunocompromised, and intensive care unit patients [2, 3] causing catheter-associated urinary tract infections (CAUTI), sepsis and wound infections [4]. As on 24th January 2021, 98 *M. morganii* genomes were available in the NCBI Genbank, of which 20 were complete genomes. The objective of this study is to characterize a new, clinically isolated XDR *M. morganii* strain by whole genome sequencing to understand its antimicrobial resistance profile.

Here, we report a complete genome sequence of *M. morganii*, isolated from the urine sample of a male patient in 2018 at Sri Sathya Sai Institute of Higher Medical Sciences (SSSIHMS) Prasanthigram, India (14.1670 N 77.8091 E). The patient was admitted to the urology ward due to urinary and fecal incontinence and had a history of Road Traffic Accident (RTA), 1 year prior to the isolation of the strain. The isolate was identified as *M. morganii* by MALDI-TOF MS. Antibiotic Susceptibility Testing (AST) and Minimum Inhibitory Concentrations (MICs) were determined using Vitek2 as per CLSI guidelines [5].

Whole genome sequencing of *M. morganii* SMM01 was performed using Illumina HiSeq X (short reads technology) and Nanopore MinION (long reads technology) platforms. The reads from both the sequencing platforms were used to generate hybrid assembly using Unicycler. To the best of our knowledge, this would be the first complete genome sequence of *M. morganii* from India.

## Data description

Upon isolation and strain purification, the isolate SMM01 was cultivated in LB broth. AST was performed using N281 card in Vitek2 and the study isolate SMM01 was found to be resistant to all the tested antibiotics except aminoglycosides (Amikacin and Gentamicin). Total genomic DNA was extracted using Macherey Nagel Nucleospin® DNA extraction kit as per manufacturer’s instructions.

Oxford Nanopore Technologies (ONT) Minion sequencing libraries were prepared using the ligation sequencing kit (SQK-LSK109) and data was collected from the FLO-MIN106 flow cell. Base-calling and demultiplexing was done using Albacore v2.0.1. MinION sequencing run produced 30,881 reads with the mean read quality score of 7.7 as assessed with NanoStat [6] (Data file 1) [7]. The passed reads were taken for adapter removal using Porechop v0.2.4 (https://github.com/rrwick/Porechop). Illumina sequencing libraries were prepared using the NEBNext Ultra II DNA library preparation kit (E7645S). The libraries were pooled after performing quantity and quality checks using Qubit2 and Agilent Bioanalyzer DNA 100 kit. Illumina HiSeq X was used to sequence the multiplexed libraries. Demultiplexing was performed using bcl2fastq v2.2 (RRID:SCR_015058). Quality of the reads was assessed with FastQC [8] and MultiQC [9] (Data file 2) [10]. The processed reads from both Illumina and Nanopore were used to generate hybrid assembly using Unicycler v0.4.8 [11] and the final assembly quality was assessed with QUAST [12] (Data file 3) [13].

The final complete genome assembly (Data set 1) [14] has a total length of 39,30,130 bp, GC content of 51.0% and genome coverage of 189.69x. A total of 3777 genes were predicted by NCBI Prokaryotic Genome Annotation Pipeline (PGAP) v 4.13 [15] in the genome. These include 3617 protein-coding genes, 78 tRNAs, 18 rRNAs, 4 ncRNAs, and 60 pseudogenes. Genome completeness analysis with BUSCO v3.0.2 [16] using the “gammaproteobacteria_odb9” dataset with 452 benchmarking universal single-copy orthologs (BUSCOs) showed the presence of 100% complete BUSCOs in the hybrid assembly (data file 4) [17]. The genome was found to possess several antibiotic resistance genes, secondary metabolite gene clusters and prophages.

Given the quality control measures applied, we believe the complete genome of *M. morganii* strain SMM01 represents a high-quality dataset that would enhance the study of the antimicrobial resistance patterns. It may further aid in comparative genomic analyses of this emerging pathogen along with its biosynthetic and metabolic potential.

Please see Table 1 for links to Data files 1–4 and Data set 1.

**Table 1:** Overview of data files/data sets

Label	Name of data file/data set	File types (file extension)	Data repository and identifier (DOI or accession number)
Data file 1	Basic quality statistics of MinION sequencing data	Portable Document Format file (.pdf)	10.6084/m9.figshare.13668881 [7]
Data file 2	Quality distribution of Illumina sequencing data	Portable Document Format file (.pdf)	10.6084/m9.figshare.13668887 [10]
Data file 3	Quast report of *M. morganii* SMM01 assembly	Portable Document Format file (.pdf)	10.6084/m9.figshare.13668890 [13]
Data file 4	Short BUSCO summary	Portable Document Format file (.pdf)	10.6084/m9.figshare.13668893 [17]
Data set 1	Genome assembly of *M. morganii* SMM01	Fasta file (.fna)	https://www.ncbi.nlm.nih.gov/assembly/GCF_015698325.1 [14]

## Limitations

The complete genome sequence of *M. morganii* SMM01 was generated from a hybrid assembly using Illumina and ONT technologies to ensure accuracy and completeness. Further, Unicycler autocorrects read errors and polishes (using Pilon) the assembly to ensure accuracy. Annotation and further downstream specialized analyses were performed using robust and validated bioinformatics tools and webservers. Therefore, the authors are not aware of any limitations in the data.

## Data Availability

The complete genome sequence and annotation data of *M. morganii* SMM01 described in this data note can be freely and openly accessed on NCBI database under the accession number NZ_CP063843. The BioProject and BioSample numbers are PRJNA673656 and SAMN16619592, respectively. All the data files can be freely and openly accessed on Figshare (https://figshare.com/). The version described in this paper is NZ_CP063843.1.

## Abbreviations

NRPS: Non-ribosomal peptide synthetases
T1PKS: Type I Polyketide synthase
XDR: Extensively drug-resistant
CAUTI: Catheter-associated Urinary Tract Infections
NCBI: National Center for Biotechnology Information
AST: Antimicrobial Sensitivity Test
CLSI: Clinical Laboratory Standards Institute
RTA: Road Traffic Accident
MALDI-TOF MS: Matrix Assisted Laser Desorption Ionization-Time of Flight Mass Spectrometry
LB: Luria Bertani
ONT: Oxford Nanopore Technologies
BUSCO: Benchmarking Universal Single Copy Orthologs

## References

[1] Morgan HDR, Oxon MA (1907). Report CII. Upon the bacteriology of the summer Diarrhoea of infants. Br Med J.

[2] O'Hara CM, Brenner FW, Miller JM (2000). Classification, identification, and clinical significance of Proteus, Providencia, and Morganella. Clin Microbiol Rev.

[3] Lin TY, Chan MC, Yang YS, Lee Y, Yeh KM, Lin JC, Chang FY (2015). Clinical manifestations and prognostic factors of *Morganella morganii* bacteremia. Eur J Clin Microbiol Infect Dis.

[4] Liu H, Zhu J, Hu Q, Rao X (2016). *Morganella morganii*, a non-negligent opportunistic pathogen. Int J Infect Dis.

[5] CLSI (2020). Performance Standards for Antimicrobial Susceptibility Testing. CLSI Supplement M100.

[6] De Coster W, D'Hert S, Schultz DT, Cruts M, Van Broeckhoven C (2018). NanoPack: visualizing and processing long-read sequencing data. Bioinformatics.

[7] Chanakya PP (2021). Basic quality statistics of MinION sequencing data.

[8] Andrews S, FastQC (2010). A Quality Control tool for High Throughput Sequence Data.

[9] Ewels P, Magnusson M, Lundin S, Käller M (2016). MultiQC: summarize analysis results for multiple tools and samples in a single report. Bioinformatics.

[10] Chanakya PP (2021). Quality distribution of Illumina sequencing data.

[11] Wick RR, Judd LM, Gorrie CL, Holt KE (2017). Unicycler: resolving bacterial genome assemblies from short and long sequencing reads. PLoS Comput Biol.

[12] Gurevich A, Saveliev V, Vyahhi N, Tesler G (2013). QUAST: quality assessment tool for genome assemblies. Bioinformatics.

[13] Chanakya PP (2021). Quast report of *M. morganii* SMM01 assembly.

[14] National Center for Biotechnology Information. Assembly: https://identifiers.org/insdc.gca: GCF_015698325.1; 2020.

[15] Tatusova T, DiCuccio M, Badretdin A, Chetvernin V, Nawrocki EP, Zaslavsky L, Lomsadze A, Pruitt KD, Borodovsky M, Ostell J (2016). NCBI prokaryotic genome annotation pipeline. Nucleic Acids Res.

[16] Simão FA, Waterhouse RM, Ioannidis P, Kriventseva EV, Zdobnov EM (2015). BUSCO: assessing genome assembly and annotation completeness with single-copy orthologs. Bioinformatics.

[17] Chanakya PP (2021). Short BUSCO summary of *Morganella morganii* SMM01.

